# Molecularly Imprinted Polymer-Based Electrochemical Sensors for the Diagnosis of Infectious Diseases

**DOI:** 10.3390/bios13060620

**Published:** 2023-06-05

**Authors:** Greta Pilvenyte, Vilma Ratautaite, Raimonda Boguzaite, Simonas Ramanavicius, Chien-Fu Chen, Roman Viter, Arunas Ramanavicius

**Affiliations:** 1 Department of Nanotechnology, State Research Institute Center for Physical Sciences and Technology (FTMC), Saulėtekio Av. 3, LT-10257 Vilnius, Lithuania; 2 Department of Physical Chemistry, Institute of Chemistry, Faculty of Chemistry and Geosciences, Vilnius University (VU), Naugarduko Str. 24, LT-03225 Vilnius, Lithuania; 3 Department of Electrochemical Material Science, State Research Institute Center for Physical Sciences and Technology (FTMC), Saulėtekio Av. 3, LT-10257 Vilnius, Lithuania; 4 Institute of Applied Mechanics, National Taiwan University, Taipei City 106, Taiwan; 5 Institute of Atomic Physics and Spectroscopy, University of Latvia, 19 Raina Blvd., LV-1586 Riga, Latvia; 6 Center for Collective Use of Scientific Equipment, Sumy State University, 31, Sanatornaya st., 40018 Sumy, Ukraine

**Keywords:** molecularly imprinted polymer (MIP), electrochemical sensor, infectious disease biomarker

## Abstract

The appearance of biological molecules, so-called biomarkers in body fluids at abnormal concentrations, is considered a good tool for detecting disease. Biomarkers are usually looked for in the most common body fluids, such as blood, nasopharyngeal fluids, urine, tears, sweat, etc. Even with significant advances in diagnostic technology, many patients with suspected infections receive empiric antimicrobial therapy rather than appropriate treatment, which is driven by rapid identification of the infectious agent, leading to increased antimicrobial resistance. To positively impact healthcare, new tests are needed that are pathogen-specific, easy to use, and produce results quickly. Molecularly imprinted polymer (MIP)-based biosensors can achieve these general goals and have enormous potential for disease detection. This article aimed to overview recent articles dedicated to electrochemical sensors modified with MIP to detect protein-based biomarkers of certain infectious diseases in human beings, particularly the biomarkers of infectious diseases, such as HIV-1, COVID-19, Dengue virus, and others. Some biomarkers, such as C-reactive protein (CRP) found in blood tests, are not specific for a particular disease but are used to identify any inflammation process in the body and are also under consideration in this review. Other biomarkers are specific to a particular disease, e.g., SARS-CoV-2-S spike glycoprotein. This article analyzes the development of electrochemical sensors using molecular imprinting technology and the used materials’ influence. The research methods, the application of different electrodes, the influence of the polymers, and the established detection limits are reviewed and compared.

## 1. Introduction

The application of specific conducting polymers in sensor design has been observed to create a synergistic effect, resulting in sufficient sensitivity and selectivity [1,2,3,4,5,6,7,8,9,10]. The conducting polymers, with their semiconducting properties [11,12,13,14] can be deposited over the signal transducer [15,16]. Chemical [17], electrochemical [12], enzymatic [18], and/or microorganism-assisted [19,20,21] formation methods have been applied previously for the formation of conducting polymers on the electrode. Although many natural recognition elements, such as enzymes [22,23,24], antigens [25], antibodies [2], DNA [26] and various receptors [27], in the sensor design can bind selected targets and provide advanced selectivity for biosensors, these natural recognition elements have some challenges to overcome, such as expensive synthesis, analytical instruments, skilled staff, stability only for days at room temperature, and poor compatibility with transducer surfaces. In this case, MIPs are an excellent alternative to sensors based on biomolecules [2,28], as they can be stable in a variety of conditions (pH, temperature, ionic strength, solvents) and allow inexpensive synthesis, label-free detection, and long-term storage without loss in performance (several months to years) [29]. The determination of particular biomarkers is used for the diagnosis of various diseases. The research of biological samples similar to samples of traditional Chinese medicine also faces challenges, such as matrix complexity, component diversity, and low levels of active components [30]. Commercially available sensors with validated properties are essential in healthcare systems [31]. Mustafa et al. [32], in their review article, overviewed the state-of-the-art of MIP sensor technologies in diagnostics and the application of MIPs for accessing analytes in biofluids. The complexity of biofluids (serum, saliva, cerebrospinal fluid, sweat, urine, nasopharyngeal fluid, and tears) is one of the challenges to overcome for sensitive and selective analysis. Moreover, the rational development of drugs and medical technologies also requires the sensitive determination of biomarkers. Conducting polymers are among many other polymers used in the design of MIPs [33,34,35,36], which are used for the detection of biomarkers of various diseases [32,37,38,39].

Nosocomial infections are commonly known as infections linked to healthcare. Infections were acquired during the process of receiving health care that was not present during the time of admission. Gram-negative organisms, such as *Klebsiella pneumoniae* and *Pseudomonas aeruginosa,* are among the most common nosocomial infections [40]. The *Acinetobacter baumannii* is the most clinically significant of the four *Acinetobacter calcoaceticus-baumannii* complex genospecies [41]. *Listeria monocytogenes* cause a potentially fatal foodborne disease, especially in newborn babies, elderly people, and immunodeficient patients. Human Immunodeficiency Virus 1 (HIV-1) is the most common type of HIV. With very few exceptions, untreated HIV-1 infection results in death [42]. The hepatotropic RNA virus known as the hepatitis C virus (HCV) damages the liver over time and may lead to cirrhosis, hepatocellular carcinoma, and death [43]. Severe acute respiratory syndrome coronavirus 2 (SARS-CoV-2) is the cause of coronavirus disease 2019 (COVID-19), which has had an unprecedented global social and economic impact and resulted in a significant number of fatalities [44].

This study aims to overview MIPs dedicated to the determination of protein-based biomarkers for the diagnosis of infectious diseases, such as inflammation and sepsis, nosocomial infections, and biomarkers of viruses like Dengue, hepatitis-C, HIV-1, or COVID-19.

## 2. MIP Formation Principles

**Figure 1:**
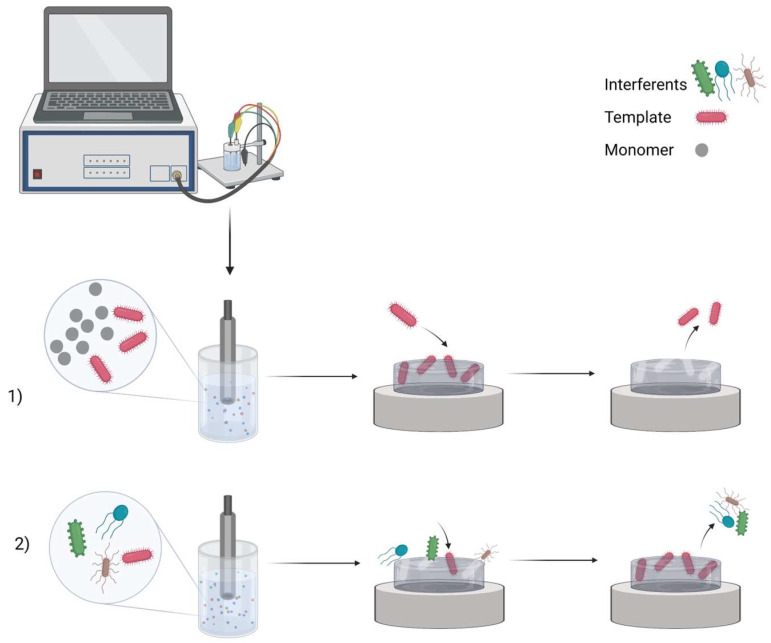
MIP preparation scheme: (1) imprinting template molecules and then washing them out; (2) rebinding process in the sample. Created with Biorender.com (accessed on 28 May 2023).

Electrochemical sensor design starts with the selection of particular electrode material. Pencil graphite electrode [45], graphite electrode [46], boron-doped nanocrystalline diamond [28,47,48], platinum electrode [49,50], gold of quartz crystal microbalance [7,51,52] or surface plasmon resonance sensor [53], etc. were used previously in the design of the electrochemical sensor. MIPs are formed from a solution containing functional monomers and an analyte, the so-called template molecule. By adjusting electrochemical parameters, electropolymerization has unique advantages over other types of polymerization methods in controlling film thickness and porosity. This method is also fast, easy, and affordable. After polymerization occurs, the template molecules are extracted from the polymer matrix to leave complementary cavities (Figure 1). Previous studies have demonstrated MIPs for low molecular weight molecules [7,28,46,47,48,52,54] or large molecular weight objects (such as proteins [49,50,55], DNA [45], viruses [56], or bacteria [57]). The development of MIPs for large objects might be somewhat challenging [2,38,58,59], and extraction of the template molecule from the polymer matrix is considered to be one of the most complex processes.

**Figure 2:**
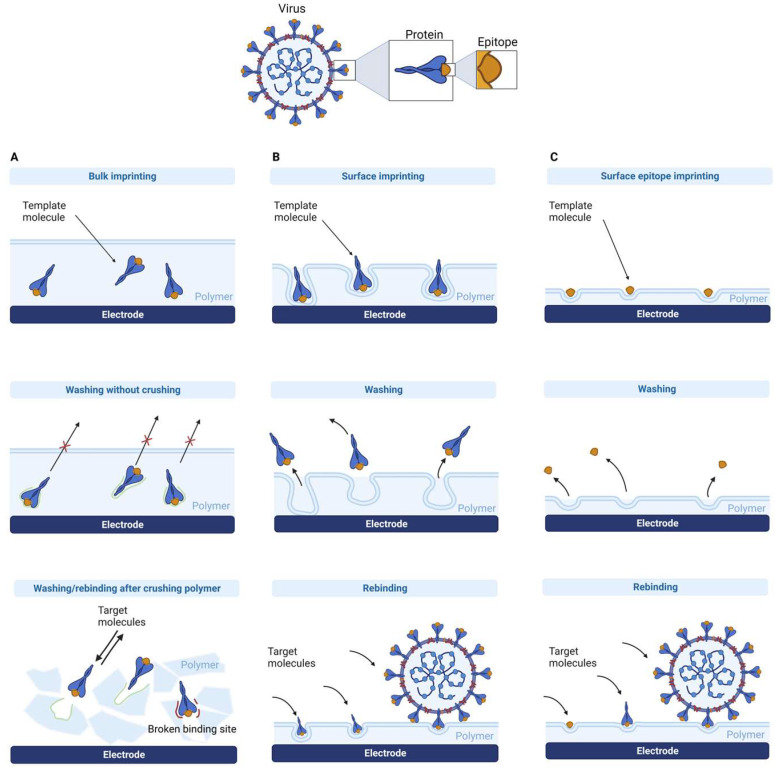
A comparison of different imprinting types: (**A**) bulk imprinting; (**B**) surface imprinting of the whole target protein, (**C**) surface imprinting of the epitope of the target protein. Created with Biorender.com (accessed on 28 May 2023).

Common imprinting techniques include bulk, particle, surface, and epitope imprinting (Figure 2). The most straightforward method for macromolecular imprinting is bulk imprinting, the traditional method that has been effective for small molecules. The advantage of this approach is that three-dimensional binding sites are formed for the entire protein; however, it has drawbacks such as diffusional limitations, template solubility concerns in organic solvents commonly used in small molecule imprinting, and conformational changes in the protein template caused by the non-physiological conditions used. To reduce diffusional limitations, bulk imprinting typically involves wet sieving or crushing of the polymer before the template removal process. Consequently, this results in polydisperse particles and could destroy binding sites [29]. In comparison, in surface imprinting, the template molecules are trapped in the surface of the polymer, and it is the most common protein and virus MIP strategy [29,60,61]. For this, a thin polymer layer or the attachment of a template to the substrate surface with subsequent imprint polymerization is used. This advantage of the easier diffusion of the large molecules into and out of the layer reduces the chance of breaking bonding sites. However, specificity may be reduced because only part of the protein is imprinted. The epitope imprinting uses a short polypeptide of the parent protein as a template during the imprinting process to recognize the entire protein. As bulk and surface imprinting face the typical problems of macromolecular imprinting, such as complex removal of the protein, conformational instability, and expensive template, epitope imprinting overcomes these obstacles using small molecule MIP principles. It also allows the use of aprotic organic solvents since polypeptides are less sensitive to environmental conditions than secondary or tertiary structures [29].

Different combinations of monomers, initiators, cross-linkers, and solvents are used to create MIPs. In aqueous solutions or organic solvents, electrically conducting polymeric structures are conducted at room temperature. Flexible MIP synthesis offers a significant benefit for imprinting biomolecules and provides an opportunity to elude conformational changes and denaturation. Although MIP-based assays have demonstrated competitiveness with assays, such as ELISA, in terms of specificity, sensitivity, and compatibility with technologies, such as lateral flow lab-on-a-chip, the commercial potential of these assays has not yet been completely realized [31]. Recently, there has been significantly increased interest in so-called point-of-care in vitro diagnostic medical devices, which need to have a long shelf life, be resistant to extreme temperature and pH changes, and have non-physiological conditions. The REASSURED criteria should be met by all new point-of-care diagnostics, including those for infectious diseases. They ought to be: “real-time connectivity”, “ease of specimen collection”, “affordable”, “sensitive”, “specific”, and “user-friendly” tests that are simple to perform and require minimal training, “rapid and Robust”, for example, to enable treatment at the first visit and not requiring refrigerated storage, “equipment-free”, “delivered” to those who need it [62]. Diagnostics at the point-of-care devices enable self-health monitoring and management [32]. Among all electrodes, screen-printed electrodes (SPEs) are one of the most suitable bases for point-of-care testing. The advantages of this approach include easy fabrication using several modifiers, including metals, metal oxides, nanocarbon materials, nanoparticles, conductive polymers, and many others. This technology also appears to be the most economical option with its convenient, fast, and versatile usage. SPEs can be used as disposable and portable electrodes due to their low cost and compactness because they contain a reference electrode, a counter electrode, and a working electrode in one small device [63]. Since non-invasive procedures do not use tools that break the skin or physically enter the body, non-invasive sampling procedures are also an important aspect of point-of-care testing, especially if they are expected to be used by the patients themselves.

## 3. MIP Application for Detection of Biomarkers of Inflammation and Sepsis

In clinical practice, a commonly used detection method for neonatal sepsis is the isolation of pathogens from body fluids. However, this method has limitations, being high cost, requiring trained personnel to run the tests, and obtaining results after a long time can lead to organ damage or death [64]. In previous research articles, the application of MIP-based biosensors for inflammation and sepsis biomarkers, such as human serum albumin (HSA) [60], C-reactive protein (CRP) [65], serum amyloid A (SAA) [66], tumor necrosis factor-alpha (TNF-α) [67], procalcitonin [68], interleukin-6 (IL-6) [69], and interleukin-1β (IL-1β) [70], was described.

### 3.1. Human Serum Albumin (HSA)

The vital plasma protein known as HSA (MW of 66.5 kDa) plays an essential role in the blood, serving as a storage and carrier for numerous endogenous (such as fatty acids and bilirubin) and exogenous (such as medications and nutrition) molecules. The reference range for HSA is 3.5–5.5 g/dL in serum and ˂25 mg/24 h in urine [71]. HSA is a sensitive sign of the risk for recurrent disease in colorectal cancer, and low serum levels of HSA have been reported to be a predictor of poor outcomes in patients with a variety of malignant tumors [72]. According to the study, low serum albumin levels are associated with an increased risk of death in patients with severe sepsis. Combining HSA and sepsis outcome scoring systems may be helpful in determining the prognosis of sepsis patients [73].

**Figure 3:**
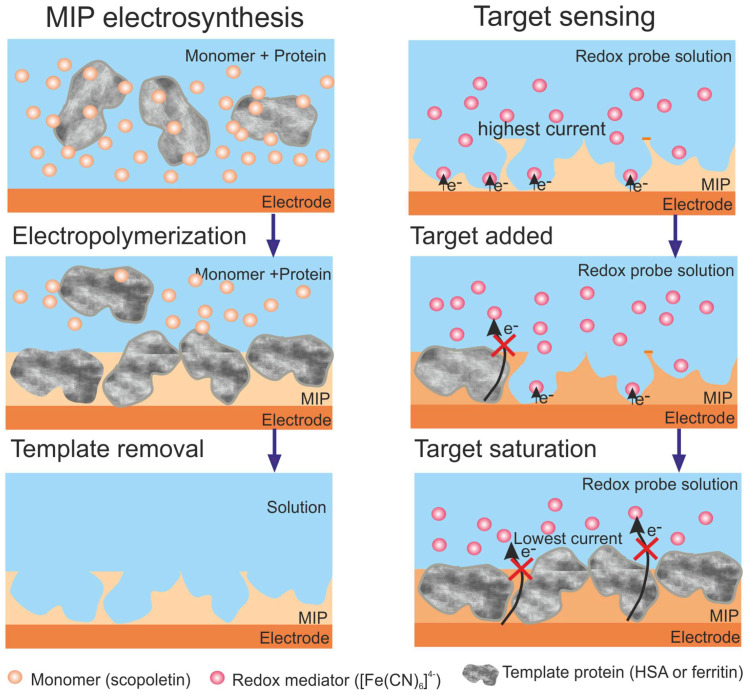
A gold electrode surface modification with surface imprinted polyscopoletin nanofilm and application of the obtained structure for the detection of HSA. Reprinted with permission from [60].

Stojanovic et al. [60] constructed an MIP-based sensor for HSA and ferritin detection in urine. Polyscopoletin was electropolymerized by imprinting HSA and ferritin separately on AuE (Figure 3). The limit of detection (LOD) indicated that the sensor was insufficient to detect ferritin in plasma (for ferritin, the LOD was 10.7 mg/L, and for HSA, the LOD was 3.7 mg/L). Fortunately, the sensor has been adapted to detect HSA in urine samples from patients with albuminuria and appears to be a viable alternative to expensive antibody testing, showing an opportunity for non-invasive clinical testing. The selectivity of the sensor toward HSA has been proven using ferritin, avidin, and lysozyme. All three proteins have been selected based on their size in comparison to HSA. Ferritin is a larger protein than HSA, with an MW of 450 kDa. Meanwhile, avidin is of a similar size as HSA, and the last protein, lysozyme, is considerably smaller in size than HSA. A selectivity test demonstrated that the proteins that are smaller in size, lysozyme and avidin, hinder the sensor response by 30%.

Zhang et al. [74] designed a sensitive MIP electrochemical sensor to detect HSA in urine by modifying AuE with gold nanoparticles and poly(thionine-methylene blue) as an electrical catalyst to improve sensor sensitivity. For the imprinting step, o-phenylenediamine (o-PD) and hydroquinone were used as functional monomers to enhance the selectivity of the sensor to HSA. A low LOD of 0.03 ng/L was achieved. L-glycine, L-glutamate, L-cysteine, L-tryptophan, L-histidine, dopamine, ascorbic acid, hemoglobin, and bovine serum albumin (two proteins with a similar molecular weight and structure to HSA) were selected for the selectivity test as the interfering molecules. The results demonstrated that hemoglobin and bovine serum albumin hindered analysis. Accurate results from non-invasive urine samples are sufficient for clinical readings and may be improved for point-of-care testing. Cieplak et al. [75] designed the first conducting MIP for HSA determination using semi-covalent imprinting. Two different functional monomers of bithiophene were used, one having a carboxyl group and the other an amino group that was then covalently linked to the corresponding functional groups of the HSA. The semi-covalent imprinting produced a LOD value of 16.6 ng/mL and an imprinting factor of over 20, confirming that well-defined locations of recognition sites were generated in the MIP cavities. The selectivity of the sensor was tested regarding such low-molecular-weight interfering molecules, such as creatinine, urea, and uric acid, with no influence on the EIS or DPV signal. Sadly, glucose’s influence on the signal was observable but solved by 1000 times dilution of blood samples. The MIP-based sensor seems promising for HSA determination in human serum samples [75].

In conclusion, all three reviewed studies for HSA used AuE with different polymers and at different sensitivities. Sensors with non-conducting poly(o-phenylenediamine and hydroquinone) polymer film showed the lowest LOD, possibly due to modification with poly(thionine-methylene blue) and gold nanoparticles for superior conductivity [74]. A novel MIP based on the semi-covalent imprinting technique and polythiophene conducting properties is worthy of further development because of the obtained imprinting factor [75]. Sensors with non-conducting polyscopoletin showed the highest LOD [60].

### 3.2. Acute-Phase Proteins

Acute-phase proteins, such as CRP (MW of ~100 kDa) and SAA (MW of ~12 kDa), are produced by hepatocytes in response to proinflammatory cytokines [76]. Brenner et al. [76] point out the main disadvantages of CRP and SAA, which are that following CRP is non-specific to inflammation and SAA, and despite it being more responsive to inflammation, there is limited evidence to use it for the prediction of the treatment.

#### 3.2.1. C-Reactive Protein (CRP)

In patients with sepsis, the nonspecific CRP biomarker CRP is a more useful tool in predicting improvement and outcome when compared to scoring systems such as the sequential score of organ failure assessment. Within two days, values in the blood can reach 300 mg/L. This value in a healthy person is usually less than 3 mg/L [77]. Therefore, CRP can be used as an early-rising biomarker for several inflammations [78]. Kumar et al. [65] developed an MIP-based biosensor to detect CRP, resulting in a LOD of 0.04 μg/mL. The analyte was imprinted in a 2-acryl amidoethyldihydrogen phosphate (AEDP) and N-(4-dimethylaminophenyl)-acrylamide (DMAA) monomer mixture on the screen-printed carbon electrode (SPCE). Both monomers were chosen to mimic the real binder of CRP—phosphatidylcholine to make the binding between the template and polymer film stronger. The AEDP phosphate group interacts with calcium ions found in CRP, whereas the positively charged nitrogen of the DMAA monomer interacts with negatively charged amino acids found in CRP. The addition of multi-walled carbon nanotubes (MWCNTs) to the coating improved the conductivity and sensitivity of electrochemical detection. The selectivity of the sensor was tested in the presence of bovine serum albumin (BSA), insulin, hemoglobin (Hb), and lysozyme as interferents. The selectivity factor of the MIP electrode was determined by analyzing the ratio between the initial linear change in the gradient of the peak current for CRP and the interfering substances that were investigated. Based on that selectivity factor, it was determined that the developed MIP-electrode was 4.9 times more selective toward CRP in a mixture of all interferents. In addition, the selectivity to CRP was even higher in the presence of BSA only, resulting in a CRP selectivity factor of 15.3. The biosensor was also used for the detection of an analyte in a human blood serum sample. Balayan et al. [79] designed a sensing platform on the SPCE using MIP technology. Such an electrochemical sensor was intended to be used for the detection of neonatal sepsis biomarker CRP. The electrode was coated with gold-platinum nanomaterials (Au-PtNMs) to increase its conductivity, and then the layer of poly(methyl methacrylate-ethylene glycol dimethacrylate) (poly(MMA-EGDMA)) with CRP imprints was polymerized. The sensor resulted in a 0.1 nM LOD. The selectivity of the sensor was tested in the comparison of biosensor activity loss percent in the presence of glucose, uric acid, ascorbic acid, acetylcholine, cholesterol, SAA, TNF-α, and procalcitonin. The results of this selectivity test are rather good since the activity loss in the presence of the interfering molecules was below 15%. The authors claim that their MIP-based CRP biosensor response time is less than 5 min, which could benefit the precocious detection of neonatal sepsis in the early stages. 

**Figure 4:**
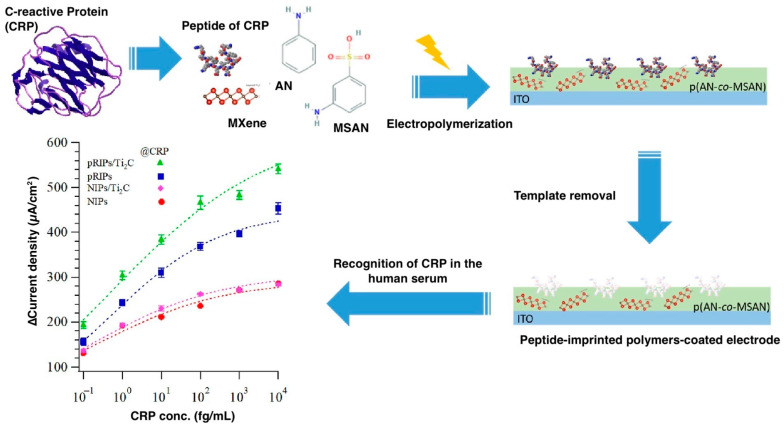
ITO electrode modification with MXene and CRP peptide-imprinted poly(aniline-co-m-aminobenzenesulfonic acid) (poly(AN-co-MSAN)). Reprinted with permission from [80].

The preparation and characterization of MXene doping CRP peptide R-imprinted poly(aniline-co-m-amino benzene sulfonic acid) coated indium tin oxide (ITO) electrodes (Figure 4) were described in a study by Lee et al. [80]. This study is interesting mainly in the application of MXenes, a relatively new class of two-dimensional inorganic compounds used for the doping of MIP. The biosensor was evaluated using cyclic voltammetry. This study demonstrated that by doping with MXene (Ti_2_C), they gained an increase in the sensing range from 0.1 to 100 fg/mL to 10,000 fg/mL. The selectivity of the biosensor was tested for several different peptides of CRP (pR, pK, pI), HSA, and egg yolk lysozyme. Further improvement could be helpful for the biosensor.

An impedimetric MIP sensor for CRP based on polydopamine with graphdiyne (GDY) as a conductive and biocompatible additive and polyethylene glycol (PEG) as an antifouling additive was described by Cui et al. [81]. The linear detection range was from 10 fg/mL to 1 µg/mL, and the LOD was found to be 4.1 fg/mL. As the competing proteins, carcinoembryonic antigen, immunoglobulin G, alpha fetal protein, and transferrin were evaluated with no evident binding on the biosensor. The feasibility of the biosensor was further tested using spiked human serum samples.

Sener et al. [68] created a microcontact imprinted biosensor for detecting procalcitonin, and the biosensor results were 99% consistent with ELISA. 

The results show that MIP is widely applicable in the determination of CRP—a biomarker of inflammation and sepsis. The application of SPE, together with MIP and test biosensors in blood samples, shows the possibility of convenient clinical testing.

#### 3.2.2. Serum Amyloid-A (SAA)

Serum amyloid A is an acute-phase protein with several isoforms; SAA1, SAA2, and SAA3 are the main ones. SAA1 and SAA2 are mainly produced in the liver, while SAA3 is produced outside the liver and is mainly found in milk but not in humans [82]. 

**Figure 5:**
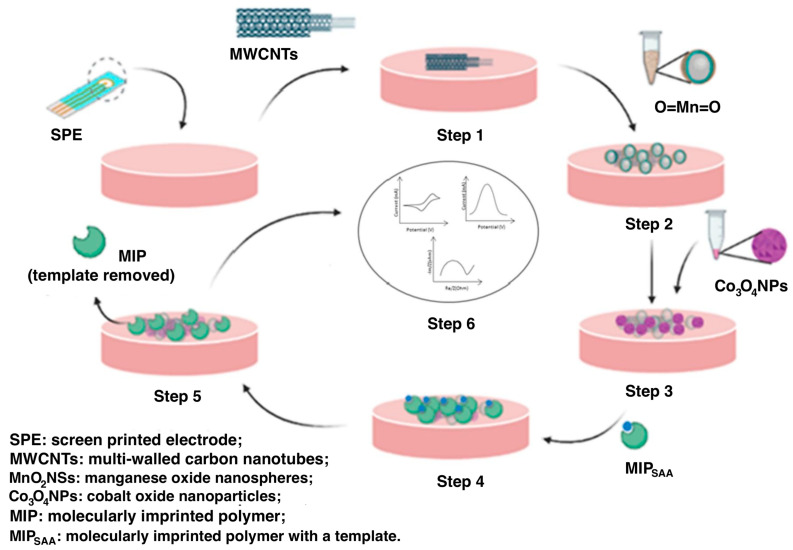
6-step SPE modification with Co_3_O_4_NPs/MnO_2_NSs/MWCNTs and SAA imprinted poly(methyl methacrylate-ethylene glycol dimethacrylate): Step 1: Electrodeposition of MWCNTs) on the bare SPE; Step 2: Modification MnO_2_NSs; Step 3: Electrodeposition of Co_3_O_4_NPs; Step 4: MIP with SAA imprints; Step 5: Removal of template from the MIP. Step 6: Application of the obtained structure as an electrochemical biosensor. Reprinted with permission from [66].

Balayan et al. [66] developed an electrochemical MIP-based biosensor for the detection of serum amyloid A as a neonatal sepsis biomarker. First, the SPE was modified with MWCNTs, manganese oxide nanospheres (MnO_2_NSs), and cobalt oxide nanoparticles (Co_3_O_4_NPs) to achieve high conductivity (Figure 5). The serum amyloid A template molecules were then imprinted in poly(methyl methacrylate-ethylene glycol dimethacrylate). An LOD of 0.01 pM (0.12 pg/mL) was achieved with a response time of fewer than 3 min. Ascorbic acid, cholesterol, glucose, uric acid, and acetylcholine were used as interfering molecules in the selectivity test. This test demonstrated that the proposed sensor was sensitive to SAA, and the loss of activity was below 12%. Unfortunately, although the authors mentioned other biomarkers of inflammation in the introduction (such as CRP, complete blood count (CBC), procalcitonin, differential leukocyte counts, and neutrophil counts), they did not use them in the selectivity test. Comparing the LOD of the developed MIP/Co_3_O_4_NP/MnO_2_NS/MWCNT/SPE electrode with other electrochemical sensors not employing MIPs for serum amyloid A detection, this biosensor is the most sensitive [83,84,85]. Accordingly, this biosensor has the potential for serum amyloid A detection.

### 3.3. Cytokines

Cytokines are a type of small protein (~5–25 kDa) that are essential in cell signaling. The effect of these small proteins may be inflammation-promoting (IL-1α, IL-1β, IL-2, IL-6, IL-8, IL-12, TNFα, IFNγ) or inflammation suppressive (IL-4, IL-5, IL-10, TGFβ) [76]. Cytokines were sought in serum and plasma samples. Several shortcomings and challenges are emphasized in terms of cytokines. Brenner et al. [76] pointed out that these cytokines are found in relatively low concentrations and, in the final result, do not help to predict the outcome of the treatment. This chapter discusses only several cytokines for which MIPs in electrochemical sensor design were published. 

#### 3.3.1. Tumor Necrosis Factor (TNF-α)

Cytokine TNF-α is a homotrimer with a molecular weight of 52 kDa. It has pleiotropic effects, growth regulation, and differentiation on various cell types. It is a key regulator of inflammatory responses and plays an essential role in inflammation, viral replication, tumorigenesis, autoimmune diseases, infections, and septic shock [86]. The electrochemical sensing platform was developed by Balayan et al. [67] for TNF-α. The SPE was modified with molybdenum disulfide nanosheets (MoS_2_NSs) and silicon dioxide-modified iron oxide nanoparticles (Fe_3_O_4_@SiO_2_NPs). Then, methyl methacrylate was electropolymerized on the electrode to imprint analyte molecules. Two-dimensional materials are highly sensitive to analytes due to their higher surface area-to-volume ratio, but they also have advantageous properties, such as flexibility, conductivity, and mechanical and thermal strength. Fe_3_O_4_@SiO_2_NPs increase the conductivity of the working electrode. The authors claimed that Fe_3_O_4_NPs are helpful in creating an environment that allows the direct exchange of electrons with the electrode, and SiO_2_ provides stability, good biocompatibility, and low toxicity. A LOD of 0.01 pM was achieved. This biosensor has a broad detection range, high specificity, rapid detection in less than 3 min, a low voltage requirement, a small sample volume, and high sensitivity. It should be noted that the sensor has the potential for TNF-α detection.

#### 3.3.2. Interleukin-6 (IL-6)

IL-6 is a pleiotropic cytokine (MW of 20 kDa) produced in response to tissue damage and infections [87]. IL-6 is an essential cytokine during the acute-phase reaction in response to inflammation and sepsis. The normal serum concentration of IL-6 is <5 pg/mL. Serum IL-6 levels were found to rise within the first hour of the infectious stimulus and rapidly peak within 2 h. In patients with sepsis, IL-6 values were found to be elevated persistently, usually >500 pg/mL. The level of IL-6 usually increases earlier than that of PCT, CRP, and fever [88]. In a meta-analysis, IL-6 was found to be a reliable biomarker of neonatal sepsis [89].

It was found that interleukin-6 serum levels appear to be a useful prognostic biomarker in patients with a diagnosis of COVID-19 pneumonia, and greater than 35 pg/mL of interleukin-6 are associated with an increased risk of mortality, mechanical ventilation requirements, and increased severity of SARS-CoV-2 induced pneumonia [90]. In another study, a binary logistic regression, the interleukin-6 level was the most significant predictor of the non-survivor group when compared to age and C-reactive protein, showing that interleukin-6 correlates with respiratory failure [91]. Currently, ELISA and Western blotting are the gold standard methods for interleukin-6 detection. However, these techniques are time-consuming and require sophisticated instrumentation [69]. 

Gonçalves et al. [69] designed an electrochemical point–of–care MIP biosensor for interleukin-6 detection, using Py and Py-COOH as a polymer matrix on an SPCE. To improve the molecular interaction between MIP active sites and the target analyte, one of the strategies is to combine conducting monomers with organic functional groups, such as carboxylic acid. Carboxylated polymers allow more reactive cavities for bonding via –COOH groups. The carboxylic groups of aniline and carboxylaniline copolymers are mainly responsible for capturing the target molecule via non-covalent interactions, such as electrostatic interactions and hydrogen bonding. Oxygen-containing groups (carboxyl (–COOH), carbonyl (–CH=O), and hydroxyl (–OH)) offer a particular environment favorable for the recognition and attachment of analytes [2]. As a result, the LOD was found to be 0.02 pg/mL in the spiked serum samples. The results showed that the developed biosensor can be effectively used for the determination of interleukin-6 with good sensitivity. This MIP biosensor is easily constructed at a low cost; the method is rapid and accurate and offers a low LOD sufficient for Interleukin-6 detection at normal and elevated levels. 

In an analytical electrochemical method for the detection of cytokine IL-6 proposed by Oliveira et al. [92], the minimally invasive manner of sampling is integrated with testing that can be used as either point-of-care, real-time, or continuous monitoring testing. The LOD of IL-6 on such transdermal sensors was as low as 1 pg/mL by sampling the cytokine on the artificial skin. Sampling and testing on the same electrochemical sensor were obtained due to the elegant combination of an array of metalized pyramidal microneedles of polycarbonate that were coated with an MIP obtained by electropolymerization of the monomer 3-aminophenylboronic acid (APBA).

#### 3.3.3. Interleukin-1β (IL-1β)

IL-1β is a pro-inflammatory cytokine (MW of ~17 kDa [70]), required for host defense responses to infection and injury. Infection with *Staphylococcus aureus,* as well as infection of macrophages with *Salmonella typhimurium*, *Shigella flexneri*, *Legionella pneumophila*, and *Pseudomonas aeruginosa,* all induce IL-1β secretion [93]. Cardoso et al. [70] created an MIP-based biosensor to target the protein IL-1β for the first time by electropolymerization of Eriochrome Black T (EBT) monomers on SPCEs. The carbon support was first modified with poly(3,4-ethylenedioxythiophene) (PEDOT) to improve the biosensor’s electrical response and stability. A modification was then applied with 4-aminothiophenol, which acted as a linker due to the -SH bonds, which can promote its binding to PEDOT, and an aromatic amine, which can be involved in the polymerization of the EBT MIP layer. A LOD of 1.5 pM was achieved in PBS, and the biosensor showed good selectivity in spiked serum samples. Myoglobin (Myo) and Immunoglobulin G (IgG) were tested as interfering molecules. This test demonstrated that Immunoglobulin G hinders the analysis more than myoglobin. To sum up, the biosensor showed relevant results and could be a simple and fast portable platform for the determination of IL-1β. 

An electrochemical impedimetric sensor on SPCE for the detection of trace cytokine IL-1β was designed with the layered composition of poly(o-phenylenediamine) (PoPD) as the underlayer and upper layer of poly(chromotrope 2R) with IL-1β imprints [94]. Such an impedimetric sensor with a layered composition allowed for obtaining an LOD of 0.23 pg/mL. The proposed sensor demonstrated good selectivity concerning three other cytokine proteins (i.e., IL-6, TNF-α, and IL-1α).

#### 3.3.4. Interleukin-2 (IL-2)

IL-2 is a 15 kDa protein, also called the T-cell growth factor, which plays an essential role in T-cell biology and can promote T-cell-dependent immune responses. Piloto et al. [95] described a combination of biorecognition elements based on 3-mercaptopropionic acid (MPA), methacrylic acid, and N,N’-methylenebis(acrylamide) as an imprinted polymer with cadmium telluride (CdTe) quantum dots for sensing purposes for the detection of cytokine IL-2. Later, this combination was used as a fluorescence quenching sensor. The sensor was applied for the detection of IL-2 in a 1000-fold diluted synthetic human serum with a LOD value of 5.91 fg/mL. Such LOD values are entirely sufficient for applications in practice since IL-2 values in cancer diagnosis are in the range of 9.4 to 19.2 pg/mL.

A summary of the MIP application for the detection of biomarkers of inflammation and sepsis is given in Table 1 and includes information on biomarkers, preparation of the MIP sensor (polymers used for MIP production, electrodes, and extraction of the template), and information about analysis (electrochemical analysis methods, LOD, LOQ, LR, and interferents).

According to the reviewed research articles, MIP-based biosensors can be applied for inflammation and sepsis biomarkers, such as human serum albumin, C-reactive protein, serum amyloid A, tumor necrosis factor-alpha (TNF-α), procalcitonin, IL-6, and IL-1β. These biosensors have advantages over traditional methods in that they provide accurate results, low costs are required, and they do not depend upon professionally trained specialists to perform them and are promising because the rapid diagnosis of sepsis is one of the most important criteria.

## 4. MIP Application for the Detection of Biomarkers of Infectious Diseases

Infectious diseases can be detected and identified using MIPs, which are imprinted with whole viruses or specific proteins—biomarkers. Simple detection of the virus can be achieved by whole virus surface imprinting because viruses are easily identified by their morphology and surface properties [37]. Other imprinting techniques and related sensitivity of the prepared MIP-based sensors are bulk imprinting, soft lithography, self-assembly, and the particle core-shell (template immobilization technique) [96]. Using MIP-based technology, viruses can be detected by a whole virus, as in the case of the Japanese encephalitis virus imprinted in the tetraethyl orthosilicate [97] or hepatitis A virus imprinted in polydopamine (PDA) [98], virus aptamer (e.g., HIV-1 gene imprinted in poly(o-phenylenediamine on ITO) [99], main protein (e.g., spike protein [49] or NS1 (non-structural protein 1—a specific and sensitive biomarker for dengue virus infection) [100] or HIV-p24 (human immunodeficiency virus p24) [101]), epitope (e.g., glycoprotein 41, gp41 (of related protein to human immunodeficiency virus type 1 (HIV-1)) [102]) templates. Surface-imprinted polymers are often used to detect viruses. However, they have high electrical resistance and are not appropriate for producing an electrochemically sensitive layer. The analytical signal (current density, resistance) of such electrochemical biosensors can be tuned by adding some additives, e.g., nanocarbon compounds or other conductive materials, to these surface imprinted polymers. Because of its high binding capacity, quick mass transfer, and efficient template removal, surface imprinting is a popular choice among epitopes and microcontact imprinting procedures. 

### 4.1. HIV-1

Three gp120 exterior envelope glycoproteins (MW of 120 kDa) are joined noncovalently to three gp41 (MW of 41 kDa) transmembrane molecules to form the HIV-1 viral spike. gp41 is a transmembrane protein that has drawn much interest as a possible target for HIV vaccines because it comprises numerous locations necessary for the infection of host cells within its ectodomain. gp41 mediates fusion between viral and cellular membranes [103]. Regardless of subtype, there are numerous techniques of HIV infection detection, depending on the illness stage. These include viral load-based detection, CD4+ cell count-based detection, antibody-based detection, antigen-based detection, and antibody-based detection. Standard techniques for determining HIV include polymerase chain reaction, ELISA, and western blotting, but because of their technical complexity, expense, and labor requirements, the majority of conventional diagnostic tests for HIV detection are not suitable for point-of-care testing. Reverse transcription-quantitative polymerase chain reaction, reverse transcriptase, and the ultrasensitive p24 assay have all been developed to cut costs but are also incompatible with the point-of-care testing [104]. To improve survival, it is crucial to identify HIV early and follow subsequent prophylactic steps.

Lu et al. [102] developed a biosensor for the detection of protein gp41 related to HIV-1 based on imprinting of the epitope in PDA. Some advantages of epitope imprinting over whole protein imprinting include easier and cheaper template acquisition, more control in template conformation maintenance, easier template removal, and recognition site accessibility [105]. The mixture of dopamine as a functional monomer and the synthetic peptide fragment 579–613 of gp41 as a template was coated on a quartz crystal microbalance chip. This QCM chip was able to bind the most templates when polymerized using 5 mg/mL of dopamine. Lower rebinding and subsequently lower sensitivity would result from a thicker film. The MIP was capable of recognizing not only the epitope template but also the entire gp41 macromolecule. The LOD of gp41 was reached at 2 ng/mL and was successfully used to monitor HIV-1 gp41 in human urine samples. This simple MIP method demonstrates how imprinted epitopes combined with CPs can be used for rapid biomolecular analysis.

**Figure 6:**
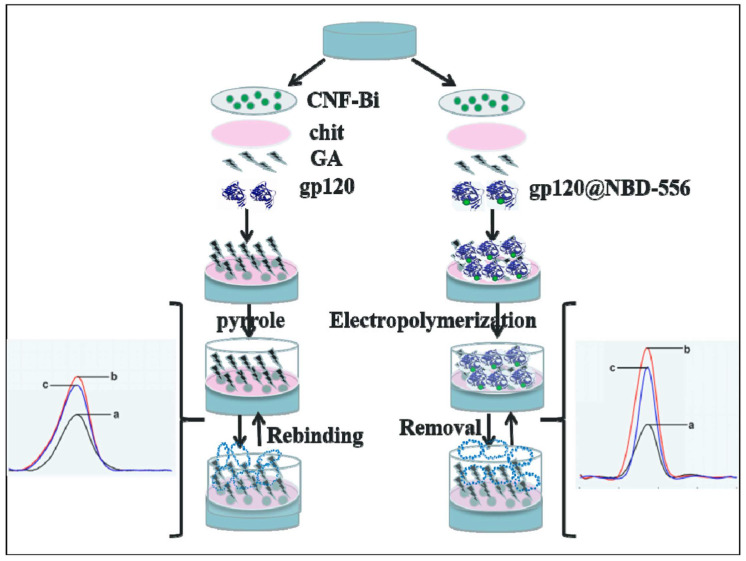
A glassy carbon electrode decorated with grapheme-like carbon nanofragment (CNF) and bismuth oxides composites (CNF-Bi) and modified with NBD-556 and gp120 conjugates NBD-556@gp120 imprinted polypyrrole as an electrochemical gp120 biosensor. Reprinted with permission from ref [106].

Ma et al. [106] developed a sensitive electrochemical MIP biosensor constructed with conducting polymer Ppy for the detection of HIV surface protein gp120 (Figure 6). Through an imine bond formed between glutaraldehyde’s aldehyde groups and the amino groups of the protein, template molecules were covalently joined to the glassy carbon electrode surface. As the free gp120 protein is conformationally unstable, its detection can be challenging. Combining free gp120 with NBD-556 (inhibitor of the interaction between gp120 and receptor) in the MIP biosensor improves recognition, linear range, and LOD of 0.0003 ng/mL by limiting the gp120 conformation. Compared to MIP with free gp120 imprinting without conjugated NBD-556, the LOD was only 0.015 ng/mL. Modifiers such as conductive carbon nanofragment and bismuth oxide composites (CNF-Bi), positively charged chitosan, and negatively charged [Fe(CN)_6_]^3−/4−^ redox probes increase current flow. The construct’s applicability was demonstrated by the accurate determination of NBD-556@gp120 in serum samples. Selectivity study showed that biosensor response to template molecules was at least two times higher than other molecules such as gp120, HIV-1 protein p24, human chorionic gonadotropin, and carcinoembryonic antigen. The development of this biosensor holds promise for the rapid detection of the HIV-1 virus for the early diagnosis of HIV and has the potential to be expanded to detect other conformationally unstable proteins.

Even though routine HIV testing of blood products has become standard, the increased transmission of transfusion-related diseases in underdeveloped nations is a result of the absence of quick, accurate, and affordable diagnostics. According to the reviewed articles, MIP-based electrochemical sensors, including whole protein or its epitope imprinting, together with CP, can be used to detect biomarkers of early HIV infection.

### 4.2. COVID-19

Severe acute respiratory syndrome coronavirus-2 (SARS-CoV-2) is a new virus that induced the COVID-19 pandemic that began in 2019. Despite this virus being rather new, it has attracted much attention from scientists. As a result, during the last few years, an increasing number of studies have been published employing MIP for the detection of SARS-CoV-2. The genome of SARS-CoV-2 encodes different proteins, such as the spike protein (each monomer of trimeric S protein is about 180 kDa) [107], the envelope protein, the membrane protein, and the nucleocapsid protein (MW of 49.5 kDa) [108,109]. These proteins can be utilized as biomarkers of COVID-19 disease. It should be noted that the spike protein is prone to multiple mutations as the virus evolves, which could limit the application of these approaches. Some infectious diseases can be detected by the whole virus or its parts. For example, coronavirus can be detected by entrapping the SARS-CoV-2 virus or its proteins, such as spike or nucleocapsid, into the molecularly imprinted polymer matrix.

Raziq et al. [110] constructed a portable MIP-based electrochemical sensor on a gold-based thin-film electrode (AuTFE) for SARS-CoV-2 nucleocapsid protein detection. Using electropolymerization, the cavities were imprinted in poly(m-phenylenediamine). LOD was achieved at 15fM. The selectivity was tested with interfering molecules, such as subunits of SARS-CoV-2 spike protein (S1), bovine serum albumin (BSA), Cluster of Differentiation 48 (CD48), and Hepatitis C virus (HCV) surface viral antigen (E2). The response to the target SARS-CoV-2 nucleocapsid protein molecule was significantly higher than to interferents. The sensor was applied to clinical samples, and it responded higher to COVID-19-positive samples. The sensors have excellent long-term stability during 9 weeks of storage. Hashemi et al. [111] created a more complex MIP based on decorated graphene oxide flakes and a mix of Ppy–boronic acid imprinted by SARS-CoV-2 antigen templates, allowing for selective and sensitive detection of the SARS-CoV-2 virus. Graphene increases the active surface area and sensitivity of sensors, and its oxidized forms have proven to be excellent candidates for biosensing applications. Combining graphene flakes with polymers improves the biocompatibility of the resulting complex, allowing for selective conjugation with target biomolecules. Boronic acid-derived compounds with a high affinity for glycoprotein structures and their use as crosslinkers could significantly improve the sensitivity and selectivity of the derived MIPs toward target templates. The selectivity was measured using several interferent molecules, such as the H1N1 influenza virus, H3N2 influenza virus, glucose, lactose, maltose, ascorbic acid, sucrose, fructose, and bovine serum albumin. This test demonstrated that the biosensor was selective for the target SARSCoV2 antigen molecule and was almost insensitive to interferences, indicating highly specific detection. All rebinding experiments were conducted in control solutions only, with no real samples used. This sensor design was successful due to a favorable low LOD of 0.326 fg/mL.

**Figure 7:**
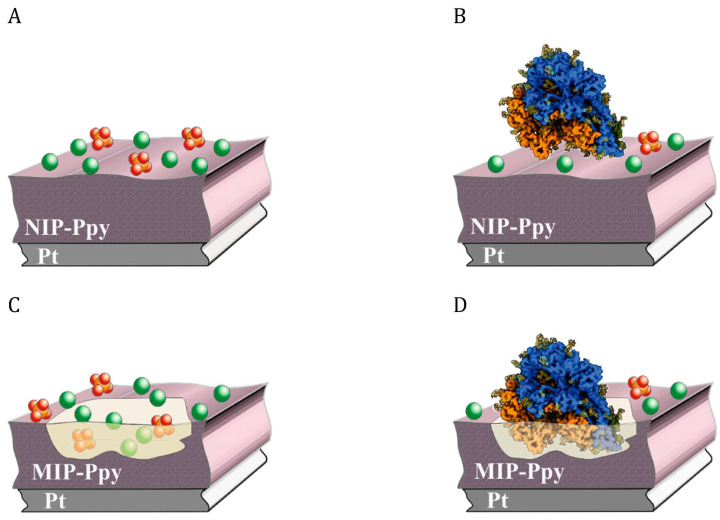
Demonstration of the interaction between NIP-Ppy or MIP-Ppy and SARS-CoV-2 spike glycoprotein and buffer solution anions (PO_4_^3−^, HPO_4_^2−^, or H_2_PO_4_^−^ and Cl^−^). (**A**). NIP-Ppy in a solution containing 0 μg/mL of SARS-CoV-2 spike glycoprotein; (**B**). NIP-Ppy in a solution containing > 0 μg/mL of SARS-CoV-2 spike glycoprotein; (**C**). MIP-Ppy in a solution containing 0 μg/mL of SARS-CoV-2 spike glycoprotein; (**D**). MIP-Ppy in a solution containing > 0 μg/mL of SARS-CoV-2 spike glycoprotein. Reprinted with permission from [50].

Ratautaite et al. [49,50] developed a sensor using molecular imprinting technology for the detection of SARS-CoV-2-S spike glycoprotein. Ppy was chosen as a CP film to entrap the template proteins. MIP (with imprinted template molecule cavities) and non-imprinted polymer (without imprinting) were synthesized on the Pt electrode (Figure 7). The results showed that the changes in MIP current are larger than in the non-imprinted polymer, and the sensor can be applied to the selective detection of imprinted SARS-CoV-2-S glycoprotein. 

Ayankojo et al. [61] designed an electrochemical MIP sensor for a quantitative study of the SARS-CoV-2 spike protein. The SARS-CoV-2 spike protein was imprinted in a thin aminophenyl boronic acid polymer film using a surface imprinting technique. Only in the presence of fluoride ions can 3-aminophenyl boronic acid be electropolymerized into a CP. The synthesis of non-conducting polymers is suggested by electropolymerization in the fluoride-free solution (aminophenyl boronic acid) [112]. Thus, aminophenyl boronic acid was electropolymerized in PBS containing NaF. Because of its water solubility and its ability to interact with amino acids, aminophenyl boronic acid is particularly valuable as a monomer. Real patients’ nasopharyngeal samples were tested, and the LOD was 4.8 pg/mL. Furthermore, the proposed sensor was compatible with a portable potentiostat and can serve as a monitoring platform for COVID-19 patients for rapid and early diagnosis. The sensor demonstrated rapid diagnostic capability with a rebinding time of 15 min and a measurement duration of 5 min.

Zhang et al. [113] developed an MIP biosensor for the SARS-CoV-2 nucleocapsid protein. The SPE was coated with gold/graphene to increase the active area and conductivity. Increasing the active area makes more binding sites available for interaction with the target analyte. This allows for greater capture of the analyte, resulting in higher sensitivity. Coating the SPE with materials, such as gold/graphene, can also improve the conductivity of the sensor. This can lead to better signal transmission and reduced electrical resistance, resulting in improved overall sensor performance. P-Arg was used as a functional monomer to produce nucleocapsid protein-imprinted cavities. P-Arg increases sensory efficiency due to its high conductivity and stability. The recovery of spiked artificial saliva and nose samples was favorable, and the stability of the sensor after a week of storage showed no significant changes.

Non-conducting polymers have also been used in several MIP-based sensor approaches of development: to detect the whole virus in saliva, an MIP-based sensor was constructed using N-hydroxymethyl acrylamide as a functional monomer and crosslinked with N,N′-methylene-bisacrylamide [56]; as a functional monomer 3-aminophenol was used in the presence of the whole SARS-CoV-2 particles to form a virus-imprinted matrix on a carbon nanotube/WO_3_-SPEs [114]; MIP-based sensor with functional monomer o-phenylenediamine for SARS-CoV-2-RBD molecule templates [115]. Also, several electrochemical biosensing approaches for detecting antibodies against SARS-CoV-2 spike proteins have been developed by immobilizing recombinant SARS-CoV-2 spike proteins on the surface of an AuE modified by a self-assembled monolayer. Cyclic voltammetry, differential pulse voltammetry, potential pulsed amperometry, and electrochemical impedance spectroscopy were chosen for the electrochemical evaluation [116,117].

In conclusion, for SARS-CoV-2 nucleocapsid protein detection, a sensor with P-Arg on the SPCE decorated with gold/graphene nanohybrids resulted in lower LOD (3 fM) [113] and wider LR than the sensor with poly(m-phenylenediamine) on AuTFE without any modifications (15 fM) [110]. This is most likely due to conductive gold/graphene nanohybrids on the electrode, resulting in more sensitive results. In addition, the low LOD of SARS-CoV-2 antigen was achieved using conducting materials such as Ppy with graphene oxide flakes; the voltammetric (LOD 0.326 fg/mL) analysis method was more sensitive than the amperometric (LOD 11.32 fg/mL) method [111]. According to the discussed articles, MIP-based electrochemical biosensors are a potential diagnostic strategy to be considered for biomarkers of SARS-CoV-2 infection.

### 4.3. Dengue Virus

An MIP-based impedimetric biosensor for dengue virus detection was developed by Arshad et al. [100]. NS1 is a non-structural protein 1 with an MW of 46–55 kDa, depending on the extent of glycosylation. Intracellular NS1 is the main protein in virus reproduction, since secreted and membrane-bound NS1 have been identified as causing the immune response [118]. It is a specific biomarker for dengue virus infection and was used as a template during the polymerization process to modify SPCE, which was subsequently coated with dopamine. To prepare the imprinting material, polysulfone nanofibers were made by electrospinning and later utilized as a support due to their substantial surface area and mechanical durability. NS1 concentrations as low as 0.3 ng/mL were selectively detected by the proposed sensor. These findings demonstrate the precision and repeatability of the developed sensor for clinical diagnosis in complex biological samples for early infection diagnosis. According to the authors of the study, the goal of achieving a specific and sensitive analysis succeeded because of the dopamine ability to self-polymerize at room temperature. This feature helped them retain the template’s exact structure (NS1). In the final results, geometrically fit imprinted sites for specific detection of the target analyte were generated. Dengue virus has been detected in other studies using MIP technology with conducting [119] and non-conducting [120,121,122] polymers. Buensuceso et al. [119] designed an MIP-based sensor on gold-coated QCM crystal for dengue NS1 detection using a terthiophene-based monomer (G03TCOOH) for epitope-imprinting. Terthiophene compounds have low oxidation potential, which makes them ideal for electrochemical functionalization and modification. A LOD of 0.056 μg/mL for NS1 protein was achieved, but the sensor was not tested in real-life samples. Overall, the sensor demonstrated long-term stability, high sensitivity, and selectivity.

In conclusion, both sensors showed satisfactory results for NS1 protein determination. However, the biosensor with PDA on the SPCE was more sensitive (LOD 0.3 ng/mL) than the epitope-imprinted sensor (LOD 0.056 μg/mL). This is probably due to the imprinting of the entire protein template and polysulfone nanofibers, which greatly increased the surface area. MIP-based biosensors can offer simple and cheap diagnostic devices instead of tourniquet tests that can be inaccurate or immunological-based tests that are time-consuming, laborious, expensive, and require complex equipment and highly qualified staff.

### 4.4. Hepatitis C Virus

**Figure 8:**
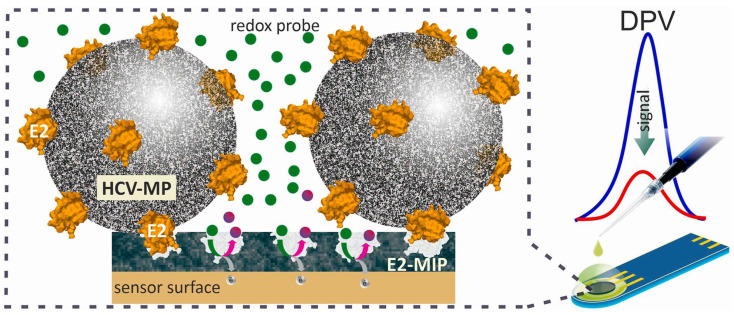
Schematic representation of HCV diagnostics principle by HCV sensor. Reprinted with permission from [123].

Antipchik et al. [123] published the first report on the development of an MIP-based electrochemical sensor for detecting the hepatitis C virus via its surface protein E2. Green fluorescent protein was used in conjunction with E2 (total MW of 54 kDa) to prevent protein agglomeration and stabilize its structure. The MIP was prepared by electrochemical surface imprinting the E2 template molecule into poly(m-phenylenediamine) on the SPE electrode (Figure 8). As the proteins are large with unstable conformation, MIP imprinting can have some challenges, such as permanent template entrapment, lower mass transfer, denaturation, and diffusion, which limits the availability of imprinted cavities. The type of imprinted polymer—surface imprinted polymer (SIP) technique—can be advantageous to overcome these challenges. In SIPs, recognition sites have excellent accessibility with better binding kinetics. However, imprinting cavities on the surface means fewer cavities overall, which may lead to less sensitivity [124]. The ability to detect both free antigen E2 (Figure 8) and the entire virus particle via E2 (Figure 8) is a clear benefit of this technique. A LOD of 0.46 pg/mL and 15 min detection time indicate that the biosensor could be used for early-stage or chronic hepatitis C detection.

Ghanbari and Roushani [125] developed a novel biosensor for hepatitis C virus core antigen by electropolymerized dopamine around the aptamer (hepatitis C virus core antigen) complex on multi-walled carbon nanotubes-chitosan modified GCE. The improved properties of the MIP-aptamer dual recognition sensor include high sensitivity, low detection limit, high stability, and high selectivity. MIP works as a filter to let only target molecules enter, and the aptamers bind with high specificity and affinity to the target molecules and identify them primarily by shape (i.e., conformation) [126]. The results demonstrated that this biosensor could be used to detect HCV core antigens quantitatively in human serum. However, no selectivity studies have been reported. Carbon nanotubes have been used as an appropriate platform in biosensors due to their high surface-to-volume ratio and electrical conductivity. Chitosan is highly adhesive, water-permeable, membrane-forming, biocompatible, and prone to chemical modification due to its reactive hydroxyl and amino functional groups. The biosensor achieved a low LOD of 1.67 fg/mL and showed high stability. Compared with electrochemical immunosensors for HCV core antigen detection [127,128], the biosensor with molecular imprinting technology showed the lowest LOD.

In conclusion, the MIP-based sensor with E2 template poly(m-phenylenediamine) showed a sufficient LOD of 0.46 pg/mL, while the sensor that used the aptamer-antigen complex in the imprinting step had more sensitive results (LOD 1.67 fg/mL), likely due to the MWCN-Chi modification and aptamer affinity for the target protein. MIP-based electrochemical sensors have great potential for the diagnosis of hepatitis C infection.

### 4.5. Nosocomial Infections

In hospitals, the most frequent cause of nosocomial infections (3–7% of hospital-acquired bacterial infections) is *Klebsiella pneumonia* (*K. pneumonia*) [40]. It can cause such conditions, including pneumonia, urinary tract infections, septicemias, and soft tissue infections. It has also developed a significant level of antibiotic resistance. *Pseudomonas aeruginosa* is the most common type of *Pseudomonas* that causes infections in human blood, lungs (pneumonia), or other parts of the body after surgery. The *Acinetobacter baumannii* is the most relevant of the four *Acinetobacter calcoaceticus-baumannii* complex genospecies, as it is the most commonly isolated in nosocomial infections with the highest mortality rates [41]. Listeriosis is a potentially fatal foodborne disease caused by the gram-positive bacteria Listeria monocytogenes. Listeriosis is most common in newborn babies, immunocompromised patients, and elderly people. Central nervous system infection and septicemia are the most common types of invasive diseases. Cases of hospital outbreaks have been reported [129,130], with an average mortality rate of 20% to 35%. The detection of an early stage of these biomarkers is lifesaving.

Sharma et al. [131] fabricated a simple MIP-based electrochemical sensor for *K. pneumonia* bacteria (rod-shaped, 2 µm long, and 0.5 µm diameter) detection using a conducting polymer Ppy (a LOD of 1.352 CFU/mL). The oxidative polymerization reaction produced positively charged Ppy, which easily captured the negatively charged bacteria inside the polymer through weak electrostatic interactions; thus, the bacteria were easily removed from the polymer matrix by sonication and rinsed with deionized water. The electrochemical sensor was tested with five interferents: two other bacteria, *Lactobacillus* and *E. coli*, different ions (K^+^, Mg^++^) and molecules (urea, uric acid) that are present in human urine. As for testing interferences, no significant change was observed in DPV current peaks, though the peak was the lowest upon contact with *K. pneumonia* bacteria showing the highest affinity to the sensor. The sensor was also tested in urine samples. Later, Pintavirooj et al. [57] created a more sensitive MIP-based electrochemical biosensor consisting of three monomers to identify *K. pneumoniae*, resulting in a high linear response with a lower LOD of 0.012 CFU/mL. Methyl methacrylate (MMA), acrylamide (AAM), and N-vinylpyrrolidone (NVP) at a ratio of 2:1:1 gave the best linearity results. The specificity for the target *K. pneumonia* was the highest compared to the other two bacteria, *E. faecalis* and *P. aeruginosa*. 

A simple MIP-based sensor consisting of a conducting Ppy layer on the ITO electrode showed efficient results for *K. pneumoniae* detection (LOD 1.352 CFU/mL) [131], but the more complex sensor consisting of three monomers mixture and graphene oxide combination on the gold SPE, it gives more sensitive detection (LOD 0.012 CFU/mL) [57].

Sarabaegi and Roushani [132] reported a sensor based on aptasensing and molecular imprinting for the detection of *P. aeruginosa* bacteria (rod-shaped, 1–5 µm long, and 0.5–1.0 µm diameter). GCE was covered with AuNPs, and then the aptamer-*P. aeruginosa* complex was immobilized on the electrode by PDA electropolymerization. The sensor showed excellent results in sensitivity and selectivity against *Shigella flexneri*, *Salmonella enteritidis*, *Escherichia coli*, and *Klebsiella pneumonia*. Results in real blood samples also showed a high (99–102%) recovery. With easy preparation, low cost, and high stability, this sensor can provide the detection of a variety of bacteria by imprinting linked aptamers, antibodies, or peptide fragments. Tokonami et al. [133] constructed an MIP-based sensor for the detection of *P. aeruginosa* using electropolymerized Ppy on the surface of Au-QCM. The characterization was obtained by di-electrophoresis. The overoxidation of Ppy allowed the formation of cavities that were shape-complementary to the template bacteria. Although the sensor had good selectivity, the LOD was significantly higher (10^3^ CFU/mL) compared to the Sarabaegi and Roushani [132] reported sensor (LOD 1 CFU/mL).

**Figure 9:**
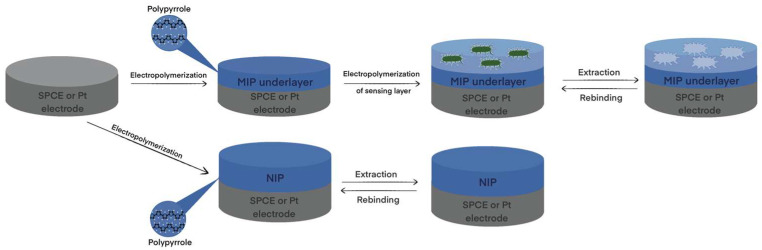
Schematic representation of platinum electrode or SPCE modification with *L. monocytogenes* bacteria imprinted Ppy [134].

Liustrovaite et al. [134] found that an SPCE electrode is more efficient than a Pt electrode for *L. monocytogenes* bacteria (0.5–2 μm-long) (Figure 9) detection resulting in a LOD of 70 CFU/mL. Since template extraction is challenging for MIP-based sensors, various extraction solutions (sulfuric acid, acetic acid, L-lysine, and trypsin) were tested. The team found that 10% acetic acid and proteolytic enzyme trypsin worked best to extract *L. monocytogenes* from the Ppy film. 

Another sensitive MIP sensor was constructed by Li et al. [135]. In this study, 3-thiopheneacetic acid was electropolymerized to entrap *L. monocytogenes* onto the GCE. This study achieved a LOD value of 6 CFU/mL. In addition, such sensors were selective against *Staphylococcus aureus*, *Vibrio parahaemolyticus*, *Shigella*, *Salmonella enteritidis*, and *Escherichia*. A more sensitive MIP-based sensor was reported by Xiaohua Jiang, resulting in a LOD of 2 CFU/mL [136]. The GCE electrode was decorated with Ti_3_C_2_T_x_ MXenes nanoribbon (Ti_3_C_2_T_x_R), and then the thionine was electropolymerized in the presence of an *L. monocytogenes* template. The sensor showed excellent selectivity results toward target *L. monocytogenes* against competing pathogens, such as *Escherichia*, *Vibrio parahaemolyticus*, *Staphylococcus aureus*, *Shigella,* and *Salmonella enteriditis*.

Since Mxenes enhance conductivity and poly(thionine) film gives superior electron transfer capability, and the sensor provides the lowest LOD of 2 CFU/mL [136] of all three discussed MIP-based sensors for *L. monocytogenes* detection. A similar but slightly higher sensitivity (LOD 6 CFU/mL) [135] was obtained using the simple construction of a glassy-carbon electrode coated with only a 3-thiopheneacetic acid layer. 

Roushani et al. [137] constructed a simple MIP-based electrochemical sensor for *A. baumannii* bacteria (1.0–1.5 μm-diameter and 1.5–2.5 μm-long) detection. GCE was coated with PDA to entrap template bacteria. The sensor showed promising results in selectivity to target *A. baumannii* against *P. aeruginosa, E. coli, K. pneumonia, S. enteritidis,* and *S. fexner*. Real blood samples were tested, and the recovery rate was 99.3 to 106.8%. The sensor provides easy preparation, low cost, and is successful in detecting *A. baumannii*.

Nosocomial infections need to be quickly identified and treated, so it is necessary to develop a rapid, specific, and easily analyzable platform to study the pathogens of hospital-acquired infections. MIP-based electrochemical sensors can be used to diagnose nosocomial infections, as they can meet the requirements of high-quality diagnostic tools.

A summary of the MIP application for the detection of biomarkers of infectious diseases is given in Table 2.

From reviewed articles about inflammation, sepsis, COVID-19, HIV-1, dengue, Hepatitis C, and some nosocomial infection biomarker detection, it can be noted that MIP technology in electrochemical biosensors for infectious diseases is well applicable. This type of biosensor could offer more potential rapid clinical or point-of-care diagnostic tests that identify specific pieces of SARS-CoV-2, HIV, dengue, etc. MIPs open up the possibility of early detection of infectious COVID-19, HIV, dengue, sepsis, and other infectious diseases. 

## 5. Conclusions

In this review, we briefly summarize recent MIP-based electrochemical biosensors for biomarkers of infection and inflammation. Different polymers have been used: polyscopoletin, polythiophene, poly(m-phenylenediamine), poly(o-phenylenediamine), polythiophene, polyaniline, polypyrrole, polydopamine, and more. Polypyrrole is still one of the most commonly used conducting polymers in the development of MIP-based biosensors. Modifications with nanomaterials, such as gold nanoparticles or multi-walled carbon nanotubes, give more sensitive results. The limit of detection results of the reviewed biosensors suggests that molecular imprinting technology can be successfully applied to detect biomarkers of inflammation: HSA, IL-6, IL-1β, IL-2, biomarkers of viral infections: HIV-gp41, HIV-gp120, Dengue-NS1, SARS-CoV-2 antigen, SARS-CoV-2 spike protein, SARS-CoV-2 nucleocapsid protein, C-reactive protein, Hepatitis C virus core antigen and E2 protein, and biomarkers of nosocomial infections: *K. pneumoniae*, *P. aeruginosa, L. monocytogenes*, and *A. baumanii*. For the diagnosis of inflammation and infections, MIP-based electrochemical biosensors have the potential to be designed as a compact, portable, disposable diagnostic platform that does not require invasive procedures, expensive medical apparatus, or professionally trained personnel to perform diagnostic tests. Epitope imprinting shows satisfactory results in epitope and whole macromolecule recognition, long-term stability, high sensitivity, and selectivity as an even more economical biosensing platform. The application of SPEs in combination with MIPs in non-invasive, complex bio-matrices such as saliva or urine opens up a huge opportunity for point-of-care medical devices that can be used by medical personnel and patients themselves. The performance of MIP-based biosensors offers attractive substitutes for natural receptors in biosensors and has a huge potential in diagnostics due to the sensitive results, easy and fast preparation, and low cost. However, most MIPs still lack high selectivity; therefore, some additional developments are required. Eventually, these developments can be based on the application of copolymers based on several different monomers; for this reason, the selection of the most optimal monomers and the elaboration of the most suitable polymerization conditions will be demanded to design efficient MIP-based structures.

## Figures and Tables

**Table 1:** Summary of electrochemical sensors based on molecularly imprinted polymers for the detection of inflammation and sepsis biomarkers.

Biomarkers	Polymers and Modifiers	Electrodes	Extraction of the Template	Electrochemical Analysis Methods	LOD, LOQ, LR	Interferents	Reference
	HSA						
HSA	Polyscopoletin	AuE	10 min in 5 mM NaOH, 5 min in 5% SDS, 10 min in 5 mM NaOH	CV	LOD 3.7 mg/L, LR 20–100 mg/L	Ferritin, avidin, and lysozyme	[60]
HSA	poly(thionine-methylene blue), PoPD, hydroquinone, AuNPs	AuE	1 mol/L NaOH, ethanol/water (*v*/*v*, 2/1) under 50 ℃	DPV, EIS	LOD 0.03 ng/L; LR 0.1–100,000 ng/L	L-glycine, L-glutamate, L-cysteine, L-tryptophan, L-histidine, dopamine, ascorbic acid, hemoglobin, and bovine serum albumin	[74]
HSA	Polythiophene	AuE		DPV, EIS	LOD 16.6 ng/mL (DPV); LR 0.8–20 µg/mL LOD 800 ng/mL (EIS); LR 4–80 µg/mL	creatinine, urea, uric acid, and glucose	[75]
	Acute-phase proteins (CRP and SAA)						
CRP	Poly(AEDP-DMAA), MWCNTs	SPCE	10% (*w*/*v*) SDS and 0.1 N HCl mixture solutions for 4 h, 0.5 M EDTA treatment for 1 h.	DPV	LOD 0.04 μg/mL	BSA, insulin, Hb, and lysozyme	[65]
CRP	Poly(MMA, Au-PtNMs	SPCE	Methanol and acetic acid (4:1) for 24 h	EIS	LOD 0.1 nM; LR 0.1 nM–500 nM	glucose, uric acid, ascorbic acid, acetylcholine, cholesterol, TNF-α, and procalcitonin	[79]
CRP	Poly(aniline-co-m-amino benzene sulfonic acid), MXene	ITO	10 mL of 5 vol% ethanol at 130 rpm for 10 min (orbital shaker), deionized water.	CV	LOD 0.1 fg/mL	pR, pK, pI, HSA, and lysozyme	[80]
CRP	PDA, GDY, PEG (antifouling additive)	GCE	Acetone for 50 min.	EIS	LOD 4.1 fg/mL; LR 10 fg/mL–1 µg/mL	carcinoembryonic antigen, immunoglobulin G, alpha fetal protein, transferrin	[81]
SAA	poly(methyl methacrylate-ethylene glycol dimethacrylate), MWCNTs, MnO_2_NSs, Co_3_O_4_NPs	SPE	Methanol and acetic acid (4:1) for 24 h	CV, DPV, EIS	LOD 0.01 pM; LR 0.01 pM–1 μM	Interferents: ascorbic acid, cholesterol, glucose, uric acid, acetylcholine	[66]
	Cytokines (TNF-α, IL-6, IL-1β, and IL-2)						
TNF-α	Poly(MMA), MoS_2_NSs, Fe_3_O_4_@SiO_2_NPs	SPE	Methanol and acetic acid	SWV, DPV, EIS	LOD 0.01 pM	glucose, acetylcholine, cholesterol, uric acid, ascorbic acid	[67]
IL-6	Ppy, Ppy-COOH	SPCE	incubation for 3 h in 0.05 M oxalic acid dihydrate, CV	EIS, CV	LOD 0.02 pg/mL; LR 0.02–2 × 10^6^ pg/mL;		[69]
IL-6	Poly(APBA)		incubation with 20 μL of proteinase K overnight at 40 °C, CV	EIS, CV	LOD 1 pg/mL		[92]
IL-1β	PEDOT, Poly(EBT)	SPCE		EIS, SWV, CV	LOD 1.5 pM; LR 60 pM–600 nM	Myo, IgG.	[70]
IL-1β	PoPD, poly(chromotrope 2R)	SPCE		EIS	LOD 0.23 pg/mL	IL-6, TNF-α, and IL-1α;	[94]

AEDP—2-Acryl amidoethyldihydrogen phosphate; DMAA—N-(4-dimethylaminophenyl)-acrylamide; MMA—methyl methacrylate; PoPD—poly(o-phenylenediamine); MWCNTs—multi-walled carbon nanotubes; PDA—polydopamine; GDY—graphdiyne; PEG—polyethylene glycol; APBA—3-aminophenylboronic acid; PEDOT—poly(3,4-ethylenedioxythiophene); EBT—Eriochrome Black T; Hb—hemoglobin; BSA—bovine serum albumin; Myo—myoglobin; HSA—human serum albumin.

**Table 2:** Summary of the electrochemical sensors based on the molecularly imprinted polymers for the detection of infectious diseases HIV-1, COVID-19, Dengue virus, hepatitis C virus, and nosocomial infections biomarkers.

Biomarkers	Polymers and Modifiers	Electrodes	Extraction of Templates	Electrochemical Analysis Methods	LOD, LOQ, LR	Interfering Molecules	Reference
	HIV-1						
gp41	PDA	QCM	5% acetic acid (in H_2_O) for five times, DI water	X-ray photoelectron spectrometer (XPS)	LOD 2 ng/mL; LR 5–200 ng/mL		[102]
gp120	Ppy, CNF-Bi, chitosan	GCE	Hyper pure water; methanol and acetic acid solution for 20 min.	CV, DPV	LOD 0.0003 ng/mL; LR 0.002–200 ng/mL	HIV-1 protein p24, human chorionic gonadotropin, carcinoembryonic antigen	[106]
	COVID-19						
SARS-CoV-2 nucleocapsid protein	PmPD	AuTFE	Ethanolic solution of 0.1 M 2-mercaptoethanol, 10% acetic acid solution	DPV	LOD 15 fM; LR 2.22–111 fM	S1, BSA, CD48, HCV, E2	[110]
SARS-CoV-2 nucleocapsid protein	P-Arg, gold/graphene nanohybrids	SPCE	Ethanolic solution containing 0.1 M 2-mercaptoethanol; acetic acid (10%) solution	DPV, EIS	LOD 3.0 fM; LR 10–200 fM	cTnI, SARS-CoV-2 spiken, HER2, BSA, CD48, MPT64	[113]
SARS-CoV-2 antigen	Ppy, graphene oxide flakes	GCE	10 vol% acetic acid, acetone, and ethanol	DPV, amperometry	LOD 0.326 fg/mL (DPV); LOD 11.32 fg/mL (amperometric); LR 0.74–9.03 fg/mL (DPV); LR 13.14–118.9 g/mL (amperometric)	H1N1 influenza virus, H3N2 influenza virus, glucose, lactose, maltose, ascorbic acid, sucrose, fructose, BSA	[111]
SARS-CoV-2-S spike glycoprotein	Ppy	Pt	Incubation in 0.05 M H_2_SO_4_ for 10 min.	Pulsed Amperometric Detection		BSA	[50]
SARS-CoV-2 spike protein	Poly(aminophenylboronic acid)	SPE	50 mM dithiothreitol for 30 min; 30 min in 10% acetic acid	SWV, CV	LOD 1.12 pg/mL; LR 0–400 fM	SARS-CoV-2 nucleocapsid protein, E2, HSA, IgG	[61]
	Dengue virus						
NS1	PDA, polysulfone fibres	SPCE	PBS; 500 μg/mL of proteinase K for 2 h in the dark	EIS, CV	LOD 0.3 ng/mL; LR 1–200 ng/mL	FBS, lysozyme	[100]
NS1	Poly(G03TCOOH), gold	QCM	Potential washing (−0.7 V) 0.1 M tetrabutylammonium hexafluorophosphate in acetonitrile	EIS	LOD 0.056 μg/mL; LR 0.2 to 10 μg/mL	angiotensin II human, glycyl glycine, bovine serum albumin, fibrinogen	[119]
	Hepatitis C virus						
HCV surface protein E2	PmPD	SPE	PBS with 50 mM dithiothreitol for 30 min, 10% acetic acid solution on vortex for 30 min	DPV	LOD 0.46 pg/mL; LR 0.01–50 ng/mL; LOQ 15.3 × 10^−5^ ng/mL	HSA, IgG, CD81	[123]
HCV core antigen	PDA, MWCNTs- Chit nanocomposite	GCE	Water, overnight in 5% *v/v* acetic acid and 1% *w*/*v* cetyl trimethyl ammonium bromide in water with stirring	CV, DPV, EIS	LOD 1.67 fg/mL; LR 5.0 fg/mL to 1.0 pg/mL;		[125]
	Nosocomial infections						
*K. pneumoniae*	Ppy	ITO	DI water, ethanol	CV, DPV	LOD 1.352 CFU/mL; LR 1–105 CFU/mL	uric acid, K^+^, Mg^++^, urea, Lactobacillus, *E. coli*	[131]
*K. pneumoniae*	Poly(MAM:AAM:NVP), graphene oxide	AuSPE	10% acetic acid for 30 min, water at 50 °C for 30 min	CV	LOD 0.012 CFU/mL; LOQ 1.61 CFU/mL; LR 101–105 CFU/mL	*E. faecalis*, *P. aeruginosa*	[57]
*P. aeruginosa*	PDA, AuNPs	GCE	Solution containing SDS 0.01 M and 5% HNO_3_ in water	CV, EIS, DPV	LOD 1 CFU/mL; LR 10–10^7^ CFU/mL	*Shigella flexneri*, *Salmonella enteritidis*, *E. coli*, *K. pneumonia*	[132]
*L. monocytogenes*	Poly(3-thiopheneacetic acid)	GCE	SDS/AA (*w*/*v*, 5%) solution	DPV, CV	LOD 6 CFU/mL; LR 10–10^6^ CFU/mL	*Staphylococcus aureus*, *Vibrio parahaemolyticus*, *Shigella*, *Salmonella enteritidis*, *Escherichia*	[135]
*L. monocytogenes*	Polythionine, MXenes nanoribbon (Ti_3_C_2_T_x_R)	GCE	0.5 M HCl	DPV, EIS	LOD 2 CFU/mL; LR 10 to 10^8^ CFU/mL	*Escherichia*, *Vibrio parahaemolyticus*, *Staphylococcus aureus*, *Shigella*, *Salmonella enteriditis*	[136]
*L. monocytogenes*	Ppy	SPCE	10% acetic acid, or sulfuric acid, or L-lysin, or trypsin	PAD	LOD 70 CFU/mL, LOQ 210 CFU/mL, LR 300–6700 CFU/mL.		[134]
*A. baumannii*	PDA	GCE	2 h in 0.01 M SDS and 10 mM HNO_3_ in water with stirring	CV, EIS, DPV	LOD (CFU/mL; LR 10^2^–10^7^ CFU/mL	*P. aeruginosa*, *E. coli*, *K. pneumonia*, *S. enteritidis*, *S. fexneri*	[137]

PDA—polydopamine, P-Arg—polyarginine, BSA—bovine serum albumin, CD48—Cluster of Differentiation 48, HCV—Hepatitis C virus, FBS—fetal bovine serum, IgG—immunoglobulin G, PmPD—poly(m-phenylenediamine).

## Data Availability

Not applicable.
